# Tailoring the Charge and pH Responsiveness of Nanoparticles by Combining LCST Smart Polymers and Polyelectrolytes on Surfaces

**DOI:** 10.1021/acs.langmuir.5c06013

**Published:** 2026-03-31

**Authors:** Buddhini C. N. Vithanage, Anthony J. DeFrancesco, Alyssa Jean Grzesiowski, Kristi Priya Choudhury, Salma Anika, Md Mahbubul Alam, Davoud Mozhdehi, Mathew M. Maye

**Affiliations:** Department of Chemistry, 2029 Syracuse University, Syracuse, New York 13244, United States

## Abstract

The modification of nanoparticles with combinations of low critical solution temperature (LCST) polymers and polyelectrolyte (PE) layers was studied. Three *n*-isopropylacrylamide-*co*-acrylamide polymers were synthesized, in which the charges could be varied from neutral to negative or positive by incorporating polyacrylic acid (pAAc) or poly-4-vinylpyridine (p4VP) units. Polymerization was centered around a disulfide initiator, which, when reduced, produced two polymer chains and a convenient linkage to gold NPs (NPs). The resulting LCST-NP conjugates possessed sharp phase changes at critical temperatures (*T*_C_), while the incorporation of pAAc or p4VP allowed for tunable charge and pH sensitivity. ^1^H NMR and FTIR spectroscopy confirmed the polymer synthesis and NP functionalization, while UV–vis and DLS were used to monitor pH and temperature sensitivity by observing changes in the surface plasmon resonance (SPR), hydrodynamic diameter (*D*_h_), and zeta potential (ζ-Pot). The isoelectric point of each system was determined, as was the relative buffering capacity of the LCST-NP, which indicated only a few LCST polymers per NP, which limited stability and charge. To overcome this, PE layers were deposited in a layer-by-layer (LbL) manner, forming PE/LCST-NP conjugates, which had layer-dependent charge and improved stability, while retaining the LCST properties of the base layer.

## Graphical abstract

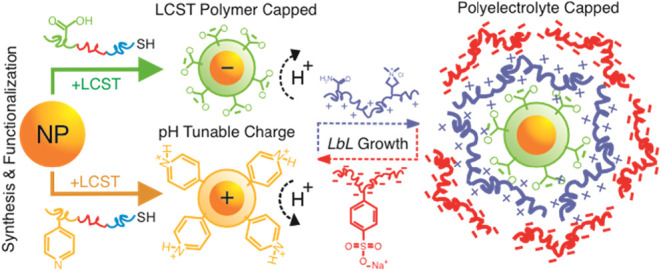

## Introduction

The ability to fine-tune the surface charge and stimuli-responsive behavior of nanoparticles (NPs) and it’ self-assembly is an important step in creating multifunctional materials with properties that can be tailored to specific applications. One original route used self-assembled monolayers (SAMs) to provide a strong surface-capping organic shell around an inorganic NP core.^1^ Nanoparticles modified or cross-linked via SAMs have responsive behavior, especially charge tunability and pH responsiveness, both in colloid,^2,3^ and thin-film morphologies.^4–7^ Another route is to employ polymers, of which there are multiple possibilities, including dendrimers,^8,9^ copolymers,^10,11^ and new bottle brush polymers.^12,13^ One way to combine the advantages of different routes is to use so-called smart polymers whose structure can be altered in response to external stimuli, which can range from temperature,^14–18^ pH,^19–24^ and light,^25–27^ to biologics,^28,29^ or biomacromolecular stimuli.^30^ One important class of these polymers is low critical solution temperature (LCST) polymers, which use a poly *N*-isopropylacrylamide (pNIPAAm) base that exhibits a reversible and sharp gel phase transition at low critical temperatures (*T*_C_).^31–34^ The LCST is tunable by copolymerization with other monomers such as polyacrylamide (pAAm).^35,36^ LCSTs are hydrophilic, where below *T*_C_, polymer chains are swollen or extended by hydrogen-bonded water,^35^ while at *T*_C_ and above, polymer chains collapse in a coil-to-globule hydrophobic phase transition triggered by the release of water, resulting in a gelling phase change or hydrogel state.^37,38^ Thus, when functionalized to bind to a surface, LCST polymers provide a convenient way to increase the hydrophilicity of a nanosystem as well as to add the function of a *T*_C_-controlled hydrodynamic diameter.

The LCST behavior of nanoconjugates has been applied in a wide range of applications, including targeted cellular uptake,^39–41^ self-assembled nanostructures,^42–46^ controlled drug release,^47–52^ catalysis,^53^ photodynamic therapy,^48,54^ and dynamic cell culture substrates.^37^ For example, LCST conjugated hollow nanocages have been used to store and then release doxorubicin,^54^ as well as to store and release imaging fluorophores.^55^ Our previous work has also broadened the use of PNIPAAm-*co*-PAAm systems to nanoparticle interfaces,^56^ as well as the codeposition of LCST polymers and single-stranded DNA (ssDNA) for DNA-encoded delivery of intercalated chemotherapy drugs.^47,57^ Ιn addition to LCST polymers for nanosystems, there is also significant interest in their use as hydrogels, especially for wound-healing applications,^58^ as well as in the engineering of biosystems that exhibit LCST or upper critical solution temperature (UCST) behavior, such as in liquid–liquid phase transitions and lipidated proteins like elastin.^59–61^

Another class of polymers used for nanosystems is polyelectrolytes (PE).^62,63^ Researchers have used PE for a number of different nanosystems,^64,65^ as well as microcapsules^66^ and biomimetic materials.^67^ One effective use of PEs is in the layer-by-layer (LbL) deposition at surfaces and NPs,^68^ which allows for the stepwise electrostatic growth of dense polymer surfaces that can be applied in a number of ways.^69^ Researchers have also studied LCST-PE mixtures, like coacervates,^70–73^ confined capsules,^74^ LCST-UCST systems,^75^ and synthesized PE hybrids that contain LCST blocks.^76^

Herein, we studied the functionalization of gold NPs with LCST copolymers modified with polyacrylic acid (pAAc) and poly-4-vinylpyridine (p4VP), which created pH-tunable negatively and positively charged systems. To increase the local charges and magnitude of the pH-tuned charges, we then deposited charged polyelectrolyte (PE) polymers in a layer-by-layer (LbL) manner on top of the LCST layer. The resulting temperature- and pH-tunable optical, hydrodynamic, and Coulombic properties were studied.

## Experimental Section

### Chemicals and Materials

Hydrogen tetrachloroaurate trihydrate (HAuCl_4_, 99.99%), trisodium citrate tribasic dihydrate (Na_3_Cit, 99.9%), *N*-isopropylacrylamide (NIPAAm, ≥ 99%), acrylamide (AAm, ≥99%), acrylic acid (AAc, 99%), 4-vinylpyridine (4VP, 95%), copper­(I) bromide (CuBr, 98%), *N*,*N*,*N*′,*N*′,*N*″-pentamethyldiethylenetriamine (PMDETA, 99%), tris­(2-carboxyethyl)­phosphine hydrochloride (TCEP, ≥98%), methanol (MeOH, ≥99.8%), and bis­[2-(2′-bromoisobutyryloxy)­ethyl] disulfide (BiBOEDS, 99.5%) were purchased from Sigma-Aldrich. Polyacrylamide-*co*-diallyldimethylammonium chloride (PADA, *M*_w_ < 100,000, 10 wt % in water) was purchased from Sigma-Aldrich, and poly­(sodium 4-styrenesulfonate) (PSS, *M*_w_ = 70,000) was purchased from Acros Organics. Ultrapure water (18.2 MΩ) was used throughout the study.

### Gold Nanoparticle Synthesis

Gold nanoparticles (NP) with a diameter of ∼10 nm were synthesized following a modified citrate reduction procedure.^77,78^ Briefly, 50 mL of a 1 mM HAuCl_4_ solution was heated to ∼95 °C for 30 min. Then, 10 mL of a 38 mM warm trisodium citrate solution was added in one aliquot to the HAuCl_4_ solution. Upon the initial color evolution to orange-red, the solution was immediately removed from heat, allowed to cool to room temperature, and stirred overnight. The approximate size and concentration of NPs were calculated based on the absorbance maximum^79^ in UV–vis spectroscopy and the related extinction coefficient (ε), which in this study was ε = 1.0 × 10^8^ L mol^–1^ cm^–1^.^77^

### LCST Copolymer Synthesis

LCST copolymers were synthesized via an atom transfer radical polymerization method,^49^ using NIPAAm, AAm, AAc, and 4VP monomer subunits. The control pNIPAAm-*co*-pAAm copolymers (**1**) were prepared using monomer molar ratios of [NIPAAm]:[AAm] = 100:0, 95:05, and 90:10. The pNIPAAm-*co*-pAAm-*co*-pAAc (**2**) and pNIPAAm-*co*-pAAm-*co*-4VP (**3**) were synthesized using [NIPAAm]:[AAm]:[AAc/4VP] = 90:05:05, unless otherwise mentioned. For example, in a typical synthesis of **2**, 1.87g of NIPAAm, 0.065g of AAm, and.066 g of AAc were dissolved in 18 mL of ultrapure water and 12 mL of MeOH for ∼30 min at room temperature in a round-bottom flask connected to a vacuum line. Next, 35 μL of PMDETA and 0.015g of BiBOEDS were added, and the mixture was frozen using liquid nitrogen and degassed under vacuum for 30 min. The flask was then filled with N_2_, and 0.010 g of CuBr catalyst was added. The mixture was then left to melt to room temperature while being stirred for ∼12h. Finally, the resulting blue solution or gel was dispersed in an additional 10 mL of ultrapure water and purified via dialysis multiple times using an 8–10 kDa membrane (Spectrum Laboratories). Approximate polymer stock solution concentrations were based on dried mass, as determined by freeze-drying a known aliquot volume of stock.^47,56^

### Nanoparticle Functionalization

To initiate the binding of the copolymer to the NP surface, the BiBOEDS disulfide linkage was first reduced by the addition of TCEP to 1 mL of the stock polymer so that [TCEP] = 100 mM. Then, 25–100 μL of **1**, **2**, or **3** was added to 1 mL of NPs so that the [polymer]:[NP] ratio was 25–100, depending on the study. The mixture was vortexed for 5 min and allowed to anneal overnight. Additional polymer could be added as needed, and the final solution also contained TCEP, which may cofunctionalize the surface. Next, excess copolymer and TCEP were removed by centrifuging the NP in a concentrated oil and decanting the supernatant at least three times, followed by redispersion in ultrapure water.

### Instrumentation

UV–visible spectrophotometry (UV–vis) was carried out using a Varian Cary100 Bio UV–vis spectrophotometer with an integrated Peltier heating controller. Heating experiments were performed at a rate of 1 °C/min. Dynamic Light Scattering (DLS) was carried out on a Malvern Zetasizer Nano ZS instrument utilizing a 173° backscattering detector and a 633 nm laser source. Zeta potential (ζ-Pot) measurements were performed on the same DLS using a zeta cell, and buffers or NaCl solutions of at least 10 mM. Proton nuclear magnetic resonance (^1^H NMR) experiments were performed on a Bruker AVANCE III HD spectrometer operating at 400 MHz with samples dissolved in D_2_O. Fourier transform infrared (FTIR) spectroscopy was performed on a Thermo Scientific Nicolet 6700 spectrometer with a LN_2_ cooled MCT detector with the sample dispersed and dried on a diamond ATR crystal. Size-Exclusion Chromatography coupled with Multi-Angle Light Scattering (SEC-MALS) was performed using a Waters Arc-HPLC system equipped with DAWN-8 multiangle light scattering (LS) and differential refractive index (dRI) detectors. Polymer samples at ∼66 μg/mL were prepared in 1× PBS and filtered through a 0.22 μm PVDF syringe filter prior to injection of a 50 μL aliquot into a Shodex OHpak SB-804 column at a flow rate of 0.5 mL/min. Data was collected and annotated using the ASTRA software, and the molar mass distributions were calculated by applying the Debye model.

## Results and Discussion

Scheme 1 shows an illustrative overview of this study. Three different types of LCST polymers based on a pNIPAAm-*co*-pAAm copolymer were synthesized utilizing a PMDETA disulfide initiator,^47,49,56^ which allowed for grafting to gold nanoparticles (NPs).

**Scheme 1:**
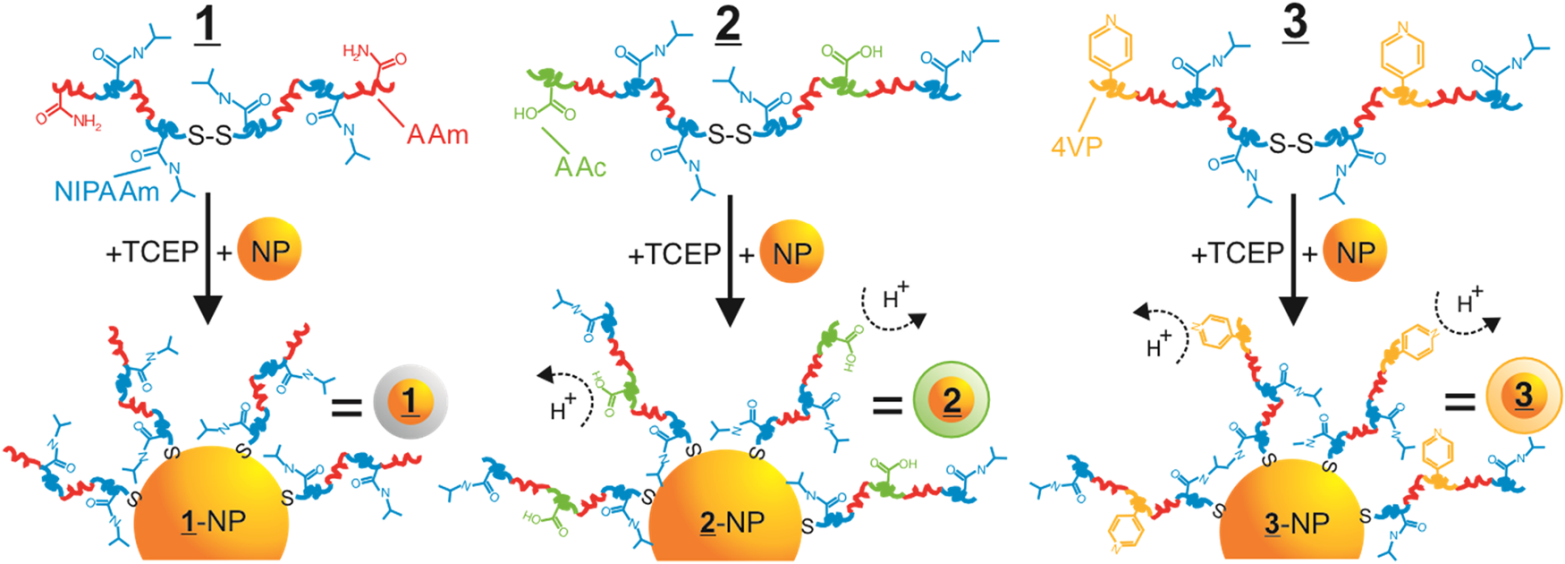
Idealized Schematic Illustrating the Three LCST-NP Systems Studied^a^

a NPs were functionalized with the synthesized pNIPAAm-*co*-pAAm (**1**), pNIPAAm-*co*-pAAm-*co*-pAAc (**2**), and pNIPAAm-*co*-pAAm-*co*-p4VP (**3**) copolymers, and the LCST-NP charge can be tuned in the negative or positive direction using pH.

The copolymers were a nominally neutral pNIPAAm-*co*-pAAm copolymer (denoted as **1**), a negatively charged pNIPAAm-*co*-pAAm-*co*-pAAc copolymer that incorporated acrylic acid (AAc) monomers (denoted as **2**), and a positively charged pNIPAAm-*co*-pAAm-*co*-p4VP copolymer that utilized 4-vinylpyridine (4VP) monomers (denoted as **3**). Here, the pH-based ionization of AAc and 4VP provides the basis for charge variations, as well as the degree of polymer hydrophobicity. We note that each copolymer synthesis used the same monomer feed ratios, total concentrations, and synthetic conditions, as described in the Experimental section. Moreover, the location and distribution of the polymer units were not controlled in this study, and the steric and hydrophobic differences between groups 2 and 3 were not considered. The **1**-, **2**-, and **3**-polymer syntheses were confirmed by UV–vis (Figure S1), ^1^H NMR (Figures S2, S3, and S4), and FTIR spectroscopy (Figures S5–S6). Each ^1^H NMR spectrum showed peak broadening and the disappearance of characteristic monomer alkene multiplets, demonstrating large molecular weights (MW) and the lack of unreacted monomers in the purified products. The FTIR spectra revealed the characteristic vibrations associated with AAc and 4VP in **2** and **3**, respectively.

Figure 1 shows the UV–vis monitoring of the LCST transition via turbidity increase as a function of temperature. The rapid increase in absorbance (i.e., turbidity) is indicative of gelation at the LCST, which is referred to as the critical temperature (*T*_C_).^26,49,50^ As described previously, at *T*_C_, the loss of coordinated water molecules causes the copolymers to undergo a hydrophilic-to-hydrophobic phase transition, leading to phase separation, gelation, and/or precipitation. The degree of polymer hydrophobicity (i.e., *T*_C_) is typically tailored by varying the [NIPAAm]: [AAm] molar ratio. For example, Figure 1a shows that **1**-polymer had *T*_C_ of 43 (i), 48 (ii), and 56 °C when prepared at ratios of 100:0, 95:05, and 90:10, respectively. For **1**, the monomer ratio is the sole way to hydrophobicity, while for the **2**- and **3**-polymers, both the synthetic feed ratio and pH can be used. Figure 1b shows the *T*_C_ of **2**-polymer at pH = 1.8–5.4. As the pH increases above the p*K*_a_ of AAc, it becomes increasingly deprotonated, and this increase in charge causes a decrease in hydrophobicity, as well as the potential for hydrogen bonding, resulting in an increase in *T*_C_. On the other hand, the **3**-polymer shows an opposite behavior (Figure 1c) because 4VP is more charged and hydrophilic at low pH. The corresponding charges of the polymers were measured using zeta-potential (ζ-Pot) measurements, as shown in Figure S7, where the **1**-polymer was nominally neutral at pH = 6.8, with a ζ-Pot = +2 mV, while the **2**-polymer was −7 mV at pH = 8.9, and the **3**-polymer was +11 mV at pH = 3.1. These charges, arising from the differences in the p*K*_a_ values of AAc (∼4.2) and 4VP (∼5.5), thus provide a convenient way to titrate LCST hydrophobicity and *T*_C_, which in turn tailors the aggregation or self-assembly properties as a function of pH (Figure S8).

**Figure 1:**
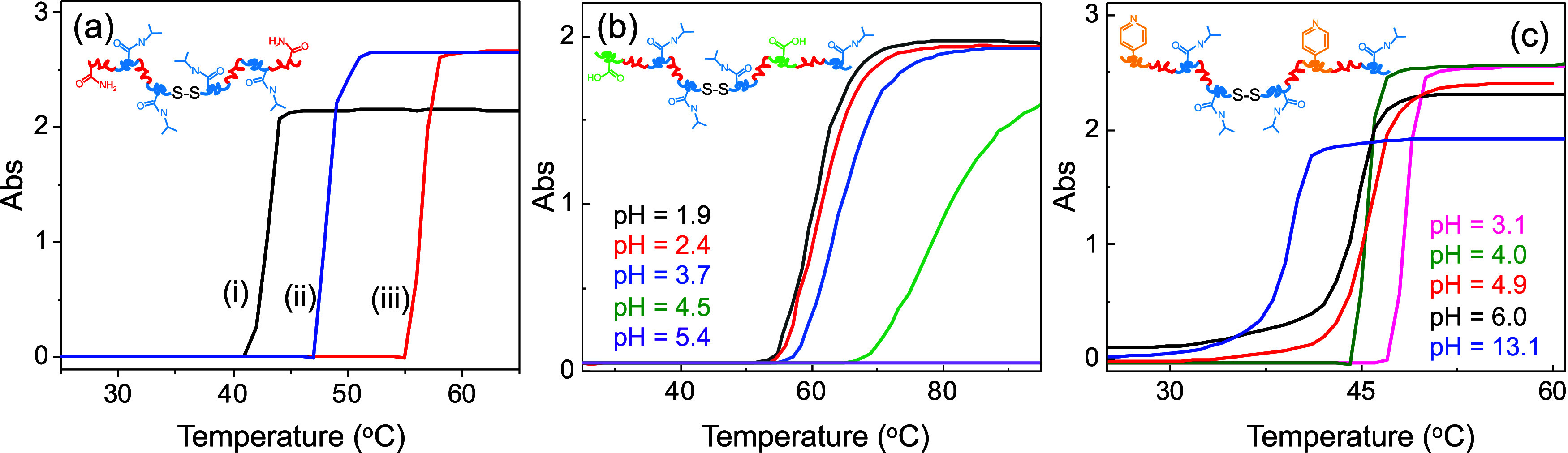
Turbidity thermal profiles of **1**-polymer (a) at [NIPAAm]:[pAAm] = 100:0 (i), 95:5 (ii), and 90:10 (iii) in pure water. Turbidity as a function of pH for **2**- (b) and **3**-polymers (c) with pH adjusted using dilute HCl and NaOH.

Next, the **1**-, **2**-, and **3**-polymers were then grafted onto NPs.^47,56^ Briefly, the polymers were freshly reduced by TCEP and then added at a [polymer]:[NP] molar ratio of ∼12.5× to an aqueous solution of NPs. After incubating overnight to allow thiol-to-gold linkage formation, the **1**-NP, **2**-NP, and **3**-NP conjugates were purified via centrifugation, redispersed, and stored in ultrapure water. Successful conjugation was confirmed by FTIR spectroscopy (Figures S5 and S6), and the spectra closely matched those of the polymers alone. The zeta-potentials of these conjugates closely resembled those of the polymers, with **1**-NP, **2**-NP, and **3**-NP having ζ-Pot of +4 mV (i), −16 mV (ii), and +15 mV (iii), respectively, as shown in Figure S7.

The conjugates also exhibited the physical properties of the polymers, as well as added pH tunability, as shown in Figure 2. Instead of macroscopic turbidity, attachment to gold NPs allows for a more sensitive measurement of the LCST by measuring the wavelength or absorbance change associated with its surface plasmon resonance (SPR) band. The SPR arises due to the collective oscillation of conduction electrons in the presence of light, indicating the changes in the local dielectric at the interface and particle coupling due to self-assembly.^80–82^ For instance, while **1**-NP showed LCST behavior similar to our previous reports (a),^47,56^ both **2**-NP (b) and **3**-NP (c) had *T*_C_ sensitivity to pH, similar to the polymers alone. When the **1**-NP conjugates were heated from 25 to 55 °C, there was a slight increase in absorbance due to the transition of the bound copolymer from the hydrophilic-to-hydrophobic form, which resides closer to the NP interface. After 15 min at 55 °C, the SPR was shifted from 520 to 580 nm, indicating NPs aggregation due to increased hydrophobicity. DLS was also used to study the aggregation of **1**-NPs (Figure S7), where *D*_h_ was ∼18 nm at room temperature and then increased to *D*_h_ ∼ 45 nm after 15 min, and *D*_h_ ∼ 110 nm after 1h, indicating aggregation. This phase transition is slower compared to that of the isolated polymers described above due to the significantly lower concentrations and fewer polymer-to-polymer interactions.

**Figure 2:**
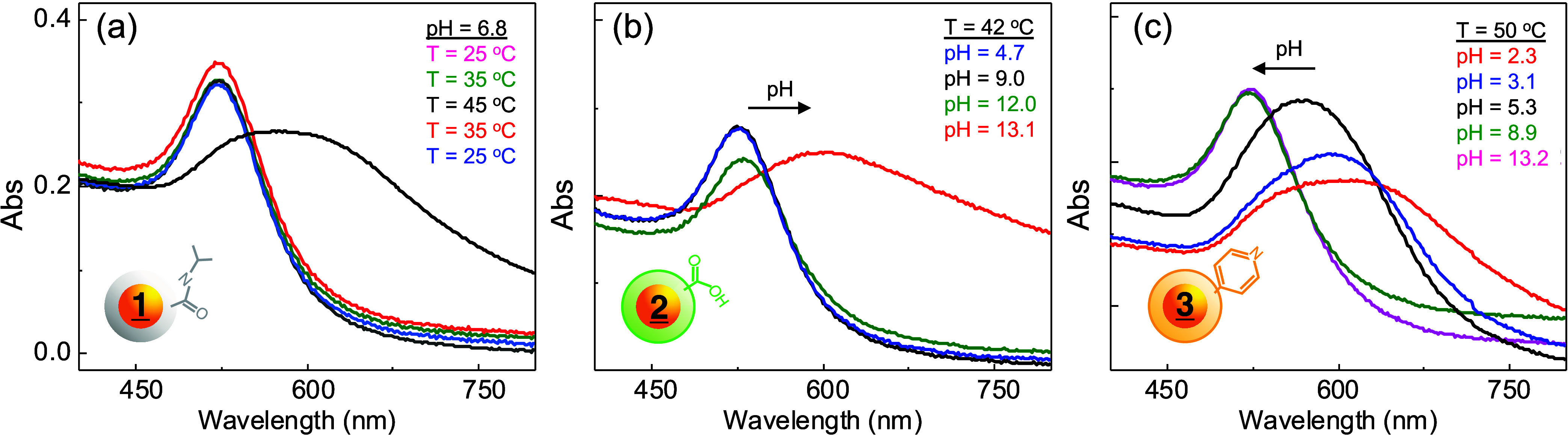
(a) UV–vis monitoring of SPR as a function of temperature for **1**-NP, where *T* was held for 5 min in pure water. (b) SPR change of **2**-NP as a function of pH at *T* = 42 °C. (c) SPR change of **3**-NP at *T* = 50 °C at the pH shown. *T* in (b, c) was chosen to best compare to **1**-NP, and the pH was adjusted using dilute HCl and NaOH, with 5 min equilibration times between *T* or pH change.

**2**-NP (Figure 2b) and **3**-NP (Figure 2c) were also studied, but were held at *T* = 42 and 50 °C, below both their *T*_C_, and the SPR change was studied as a function of pH. **2**-NP showed a lack of significant SPR redshift until pH > 12, where a decrease in absorbance was first observed, followed by broadening and a decrease in extinction, which indicated NP aggregation, closer NP-NP distances, and screening. **3**-NP, on the other hand, was stable at high pH values, but not at pH < ∼8, where aggregation was observed to a greater extent. Such changes are due to the protonation and deprotonation of the AAc and 4VP segments of **2** and **3**, respectively, which closely align with their p*K*_a_ values, as described below.

This aggregation is associated with the coil-to-globule transition of the LCST polymer as it becomes more hydrophobic. To measure this, we performed a similar pH titration of 2-NP and 3-NP and correlated the SPR change with the *D*_h_ change. For example, Figure 3a shows the UV–vis (i) and DLS (ii) monitoring of **2**-NP during a pH cycle that starts at 5.5, decreases to 4.2, and returns to 5.6. As mentioned above, the SPR redshift of Δλ ∼15 nm at pH 4.2 is indicative of a dielectric change in the shell area surrounding the NP, as well as closer NP-to-NP interparticle distances. We interpret this shift as a result of the clustering of small aggregates of only a few NPs. This is reinforced by the *D*_h_ increase from ∼26 to ∼105 nm. These physical changes were reversible, as shown when the pH was increased to 5.5, the SPR returned to λ_Abs_ = 525, and *D*_h_ = 37 nm. Figure 3b shows a similar set of data for **3**-NP in response to cycling from pH 5.34 to 10.41, and then returning to 5.10. At a higher pH, a broader SPR with a larger Δλ = 75 nm was observed (Figure 3b–i), and *D*_h_ increased to ∼150 nm. Like **2**-NP, the **3**-NP pH response was reversible, and both λ_Abs_ and *D*_h_ returned upon a decrease in pH.

**Figure 3:**
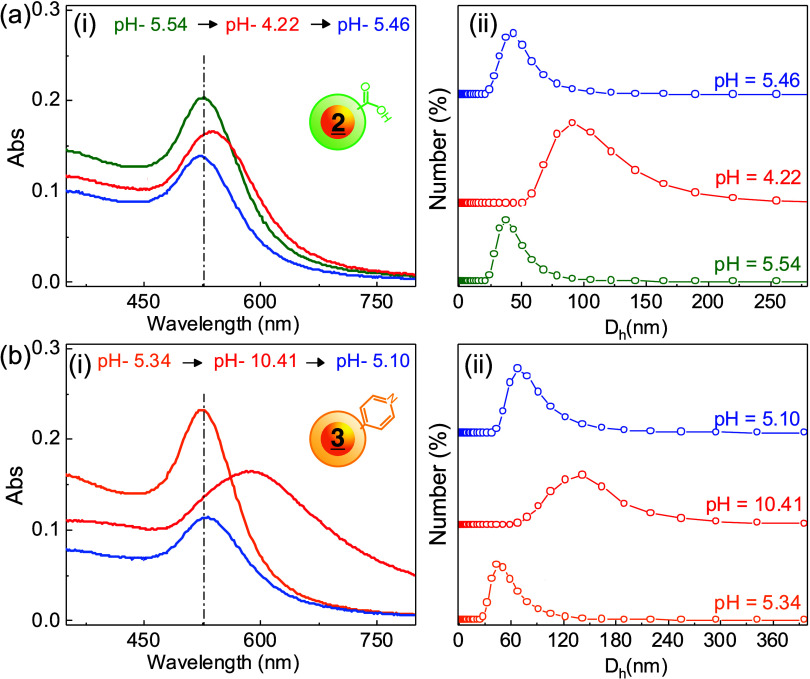
pH sensitivity of **2**-NP (a) and **3**-NP (b). SPR and *D*_h_ as measured by UV–vis (i) and DLS (ii) for the pH cycles shown at 25 °C. Concentrations were diluted during pH change by the addition of dilute HCl and NaOH, with 5 min equilibration times between measurements at *T* = 25 °C.

To better understand the charge-to-pH relationship of these systems, a systematic titration of **2**-NP and **3**-NP was carried out. First, the zeta-potential changes were measured, as shown in Figure 4. For **2**-NP (a), when fully protonated at low pH, ζ = +0.57, and as pH increased, we observed an isoelectric point (neutral charge) at pH ∼ 2.01, and when fully deprotonated at high pH, its highest charge of ζ = −22.6 was measured. As expected, **3**-NP (b) showed an opposite trend; when fully protonated at low pH, ζ = +18.2 was measured, and when the pH was increased, an isoelectric point of pH ∼ 5.46 was determined. When fully deprotonated at a high pH, ζ = – 2.79 was measured. In contrast, when fully protonated at low pH, **1**-NP had ζ = +0.11, and when fully deprotonated, ζ = +0.50 (see SI).

**Figure 4:**
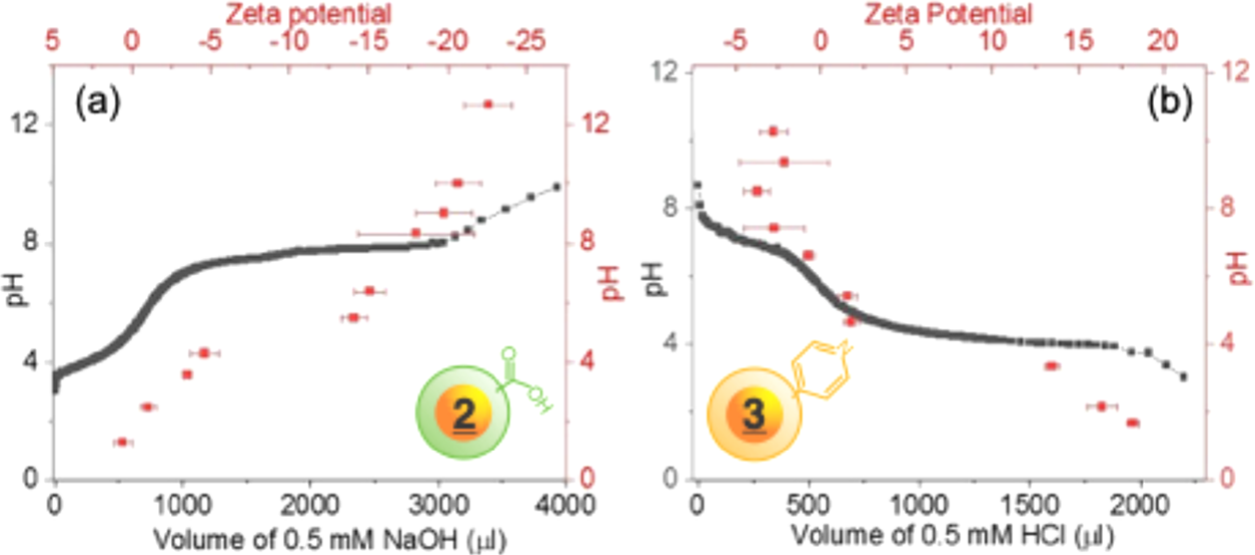
Measured zeta potential (red squares, red axis) and solution pH (black squares, black axis) during the titration of **2**-NP (a) and **3**-AuNP (b) with NaOH and HCl, respectively. [NP] = 7–9 nM, in 10 mM NaCl at *T* = 25 °C.

We next considered the buffering capacity of **2**-NP and **3**-NP in water, and Figure 5 shows the pH measurement during **2**-NP (a) and **3**-NP (b) titration using dilute NaOH and HCl, respectively. For instance, unlike the zeta-potential measurements, here, **2**- and **3**-NP were added to ultrapure water, and the resulting pH change was measured before performing titration with base and acid, respectively. When a **2**-NP solution at a concentration of ∼19.3 nM was first added to pure water, a pH of 5.10 was measured, which corresponds to the release of ∼3.51 nmol of ionized protons. As the particle was titrated with NaOH, we observed a considerable buffer region at pH ∼ 7.5 before a final increase in pH due to excess base. On the other hand, when **3**-NP was added to ultrapure water (b), the resulting pH was 6.40, which corresponds to ∼0.40 nmol of ionized protons. When titrated with HCl, two buffer regions were observed: a short one at pH ∼ 7.5 and a longer one at pH ∼ 4.8.

**Figure 5:**
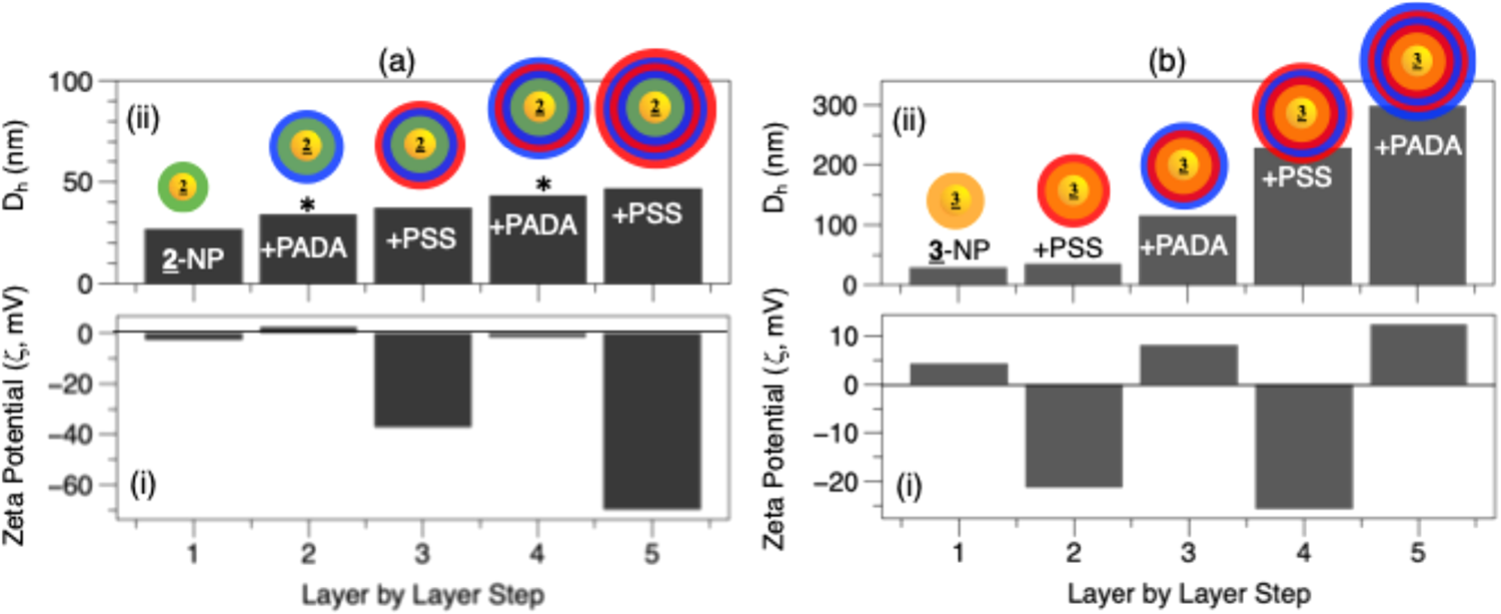
PE LbL comparisons of **2**-NPs (a) and **3**-NPs (b) at different layers by ζ-Pot (i) and *D*_h_ (ii). * indicates that DLS measurements showed aggregates of 250–300 nm (see Figures S11–S12 for the individual data sets). All samples were measured in 10 mM NaCl at *T* = 25 °C, with the pH adjusted using dilute HCl and NaOH.

Overall, these charges at the NP interface are modest, and applications ranging from drug delivery to drug encapsulation or self-assembly require higher local charges. One challenge to the **1**-, **2**-, and **3**-NPs created via LCST polymers like ours is the relatively low final polymer coverage, owing in large part to the high molecular weight (MW) and length of each polymer, which results in large footprints, resulting in a lot of open space at the interface. Our previous studies showed that reduced **1**-polymers have MWs estimated between 70,000 and 250,000 depending on the ratios and conditions,^56^ and others have reported 60,000–100,000.^54^ To estimate the MW of the **1**-, **2**-, and **3**-polymers used in this study, replicate syntheses were performed, and the MWs were determined using SEC-MALS studies. The results are presented in Table S1 and Figure S9. SEC alone measured **1** and **3** to have MW of ∼160,000, and SEC-MALS showed the primary separation fraction with an MW of 180,000–190,000. The replicate polymer **2** was shorter, with an MW of ∼90,000, as measured by both SEC and SEC-MALS.

The footprints of these long polymers can be used as an advantage when cofunctionalizing the surface, and we previously took advantage of this by filling the surface with ssDNA.^47^ Thermal gravimetric analysis (TGA) and transmission electron microscopy (TEM) were used to estimate that each NP had 8–12 **1**-polymer chains on it. Because the NP and polymers in this study were prepared using the same methodology and conditions, we assume that **2**-NP and **3**-NP have similar polymer coverage, which may or may not be correct, considering the influence of the interstrand charge repulsion of **2** and **3** during functionalization, which would result in lower coverage. In the future, one way to increase the coverage would be to employ a slow salt aging step, as done with ssDNA oligomers,^83^ which decreases electrostatic repulsion at the interface.

As an alternative approach, we explored the possibility of enhancing the colloidal stability and surface charge without losing the temperature or pH responsiveness. To this end, we added a layer-by-layer (LbL) deposition step using two common polyelectrolytes (PEs), poly­(acrylamide-*co*-diallyldimethylammonium chloride) (PADA) as the cationic PE and poly­(sodium 4-styrenesulfonate) (PSS) as the anionic PE.^84–86^

Scheme 2 shows an illustration of our LbL approach, while Figure 5a shows the zeta potential (i) and DLS (ii) measurements during LbL deposition on **2**-NP. Here, we refer to the base LCST polymer layer as layer I, which was negative for **2**-NP. Next, an aqueous solution of PADA was added (layer II) at an equivalent [PADA]:[**2**-NP] ratio of ∼100×. After incubating for 2h with stirring, excess PADA was removed by centrifugation of the PADA/**2**-NP, followed by redispersion in water and the addition of a layer of PSS (layer III), at similar equivalents, and so on until a purified five-layer PSS/PADA/PSS/PADA-**2**-NP was formed. The zeta potential showed charge reversal with each successive deposition of PE (5a-i), where the PADA layers resulted in only a minimal positive charge gain (∼5 mV), suggesting that most of the ammonium ions neutralized the surface. Meanwhile, the PSS layers resulted in charges of up to ∼−45 mV, which is a 3× increase from 2-NP alone. DLS observed an increase in *D*_h_ with each successive PE deposition, with a final *D*_h_ of ∼45 nm (5a-ii). The SPR of the NPs was also monitored after each layer and resulted in either no significant Δλ in the case of **2**-NP, or broadening of the SPR in the case of **3**-NP, as shown in Figure S10.

**Scheme 2:**
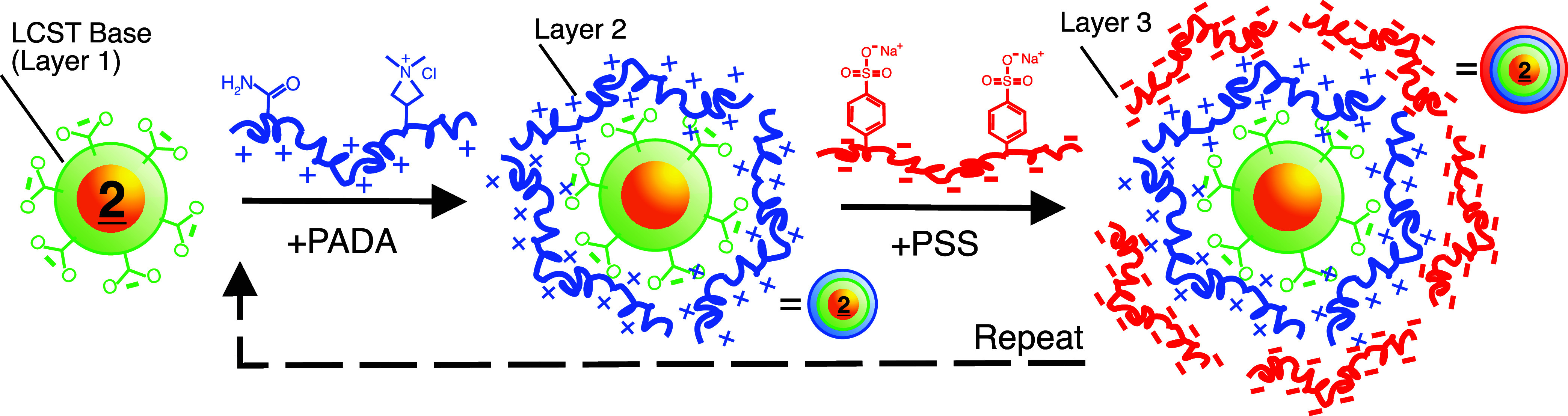
An Idealized Illustration of the Layer-by-Layer (LbL) Deposition of PADA and PSS Polyelectrolytes (PE) at **2**-NP^a^

a After layer 3, the process can be repeated as needed. The purification steps between the layers are not shown. **3**-NP is also used as a base layer, with subsequent alternating PSS and PADA depositions (not shown).

Figure 5b shows an identical procedure using the **3**-ANP base, where PSS was first deposited, followed by the deposition of PADA. Interestingly, this layering resulted in better charge alteration, with a significant positive ζ of 5 ∼15 mV (5b-i) for the PADA layers. Meanwhile, *D*_h_ also increased with each additional layer, with larger values of ∼300 nm observed after 3 layers, indicative of some clustering or aggregation of the NPs associated with the high positive charges (5b-ii).

Importantly, these PE-**2/3**-NPs were found to still retain the temperature and pH sensitivity of the base LCST layer, but with higher stability and improved cycling behavior. As shown in Figure 6, the SPR of PE-**2**-NP was monitored at pH 7 ∼ 8 (a) and 4 ∼ 5 (b) and temperature cycled. Compared to **2**-NP (layer I, Figure 6a-i), the PSS/PADA-**2**-AuNP (layer III, Figure 6a-ii) and PSS/PADA/PSS/PADS-**2**-AuNP (layer V, Figure 6a-iii) had similar temperature-dependent SPR behaviors at higher pH values, with little λ change but some variability in Abs. On the contrary, Figure 6b shows the SPR shifts observed at lower pH values for each PE-**2**-NP (i-iii), demonstrating that the LCST layer characteristics are preserved. Because the LCST behavior is retained, it can be assumed that the PE layers do not block the protonated and deprotonated ions and that LCST dehydration is not prohibited. Moreover, while SPR redshifts are observed, a rapid decrease in extinction is not, suggesting that PE-**2/3**-NPs have more colloidal stability, possibly due to higher steric or electrostatic repulsion or smaller aggregate sizes. Further, the higher layers of PE likely also enhance the overall electrostatic screening of the particles, further improving stability.

**Figure 6:**
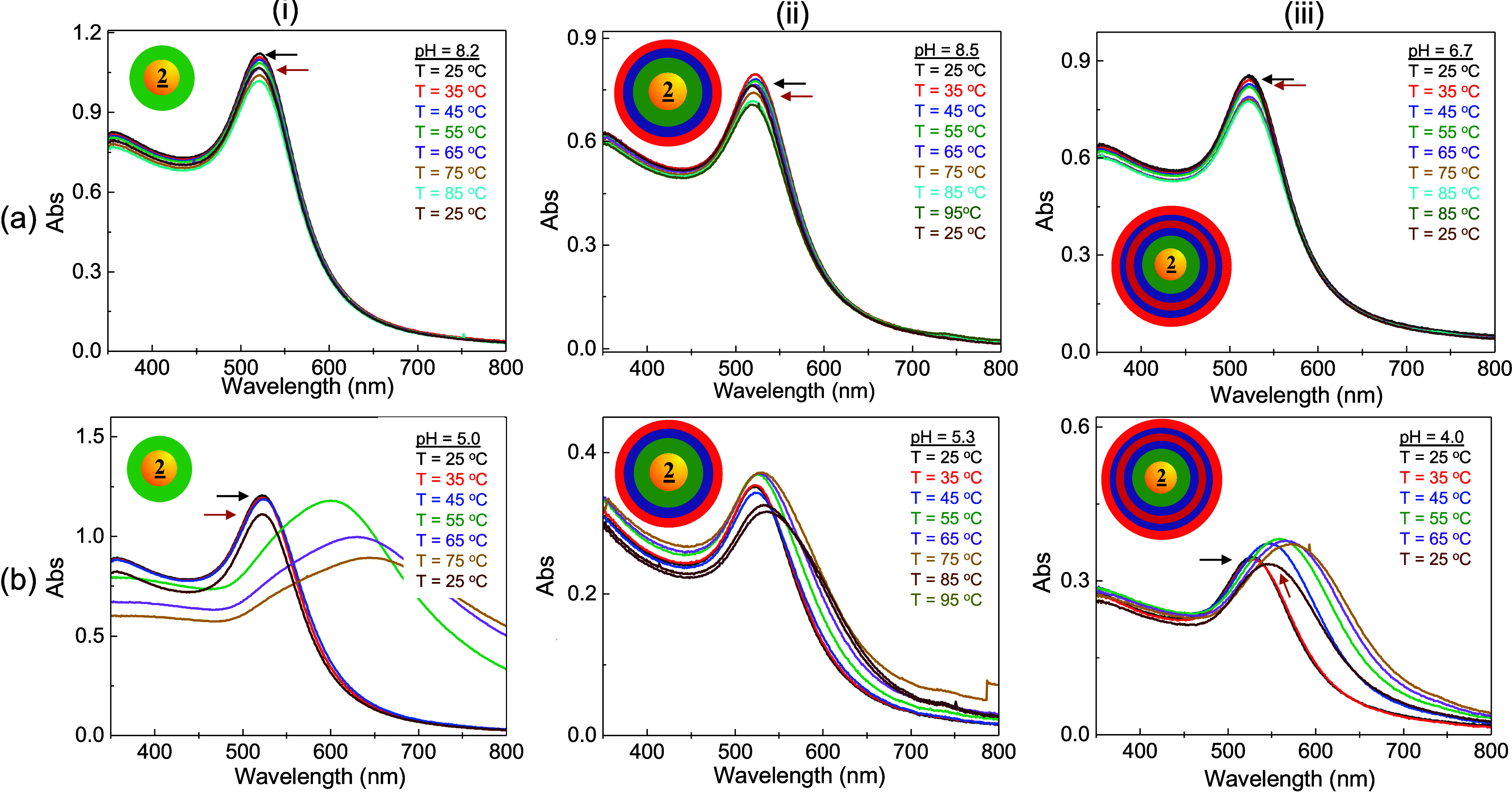
Comparison of temperature-dependent SPR behavior of PE-**2**-AuNP at high (a) and low pH (b) at the pH and T shown. The **2**-NP bases (Layer I, (i)), PADA/PSS-**2**-NP (Layer III, (ii)), and PADA/PSS/PADA/PSS-**2**-NP (Layer V, (iii)) were studied. The arrows indicate the temperature-cycling spectra. In pure water and 5 min equilibration time between T.

Taken together, these results show a strategy to create pH- and temperature-sensitive nanoparticles. While the temperature sensitivity is tailored by the monomer concentration ratios, high local charges can be fine-tuned by the monomer functionality and pH. The electrostatic addition of polyelectrolytes to the surface of the bound LCST layer provides another convenient way to tailor the charge while also providing higher stability and local densities of functional groups. The increased number of functional groups at the final periphery of PE- **2** **/** 3-NPs thus creates a convenient interface to cross-link polymers or biomaterials in the future, as well as a dense interface to craft or deposit additional nanomaterials, which is part of our ongoing work.

## Conclusions

In this work, low critical solution temperature copolymers of pNIPAAm-co-pAAm-co-pAAc and pNIPAAm-co-pAAm-co-p4VP were formed using a disulfide initiator, which provided a means of chemically grafting onto the surface of gold nanoparticles. After characterization of the polymer products, nanoparticle conjugation was confirmed by FTIR and ^1^H NMR spectroscopy, and the critical temperatures and dynamic properties of the polymer-capped nanoparticles were probed by UV–vis and DLS studies. The polymer–particle conjugates were pH-sensitive, as confirmed by nanoparticle aggregation, changes in hydrodynamic properties, and changes in colloidal charges. The isoelectric point of the polymer–particle conjugates, and the buffering capacity were measured. To increase the overall charge and stability of the conjugates, polyelectrolyte polymer layers were deposited in a layer-by-layer manner on the surface, which resulted in larger hydrodynamic diameters and tailorable and higher local charges, while maintaining pH and temperature sensitivity.

## Supplementary Material



## Notes

Polymer pH-dependent thermal profiles, ^1^H NMR spectra, FTIR spectra, Zeta Potential measurements, and details; DLS characterization of polymer control, SEC-MALS study of polymer molecular weight, UV–vis of polyelectrolyte deposition, DLS and Zeta Potential spectra of polyelectrolyte conjugates (PDF)
