# Supplementary material for: Tailoring the Charge and pH Responsiveness of Nanoparticles by Combining LCST Smart Polymers and Polyelectrolytes on Surfaces
Source: Langmuir. 2026 Mar 31;42(14):9744–53. doi: 10.1021/acs.langmuir.5c06013 (PMC13085811; doi:10.1021/acs.langmuir.5c06013)
Supplement: Supplementary file 1 [file la5c06013_si_001.pdf]

Supporting Information

Tailoring the Charge and pH Responsiveness of Nanoparticles by Combining LCST Smart Polymers and Polyelectrolytes at Surfaces

Buddhini C. N. Vithanage, Anthony J. DeFrancesco, Alyssa J. Grzesiowski, Kristi Priya Choudhury, Salma Anika, Md Mahbubul Alam, Davoud Mozhdehi, Mathew M. Maye*
mmmaye@syr.edu

Department of Chemistry, Syracuse University, Syracuse, New York 13244

Contents

Number of Pages	8
Number of Figures	12
Number of Schemes	0
Number of Tables	1

Table S1: SEC-MALS results.

Polymer	SEC-RI MW_{Ave} (Mw/Mn)	SEC-MALS MW (g)	MALS RMS Radius
**1**	1.63×10^5 (1.04)	1.86×10^5	16.3 ± 0.5 nm
**2**	8.64×10^4 (1.02)	93.2×10^4	20.3 ± 2.5 nm
**3**	1.63×10^5 (1.02)	1.94×10^5	17.0 ± 2.5 nm

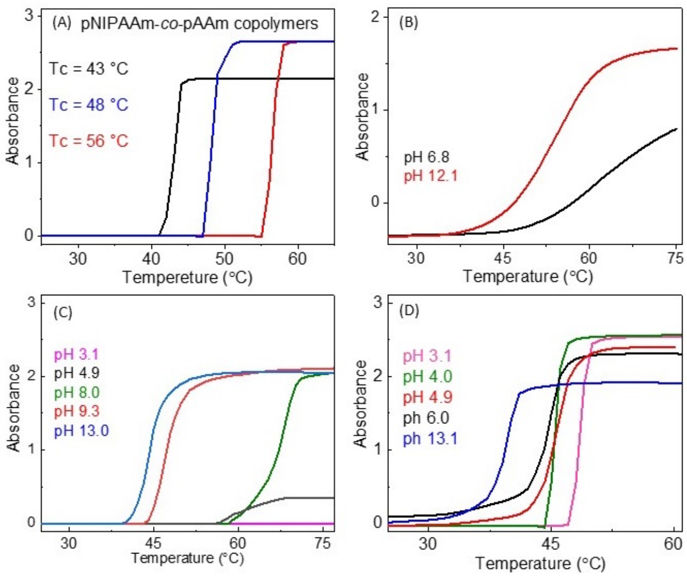

Figure S1: (**a**) Thermal profiles (1˚C/min) of polymer **1** with monomer ratios of NIPAAm: AAm 100:00 (black), 95:05 (blue) and 90:10 (red). Thermal profiles of polymer **3** with monomer ratios of NIPAAm: AAm: 4VP, 80:10:10 (**b**), 86:07:07 (**c**) and 95: 05: 05 (**d**), at the pH indicated.

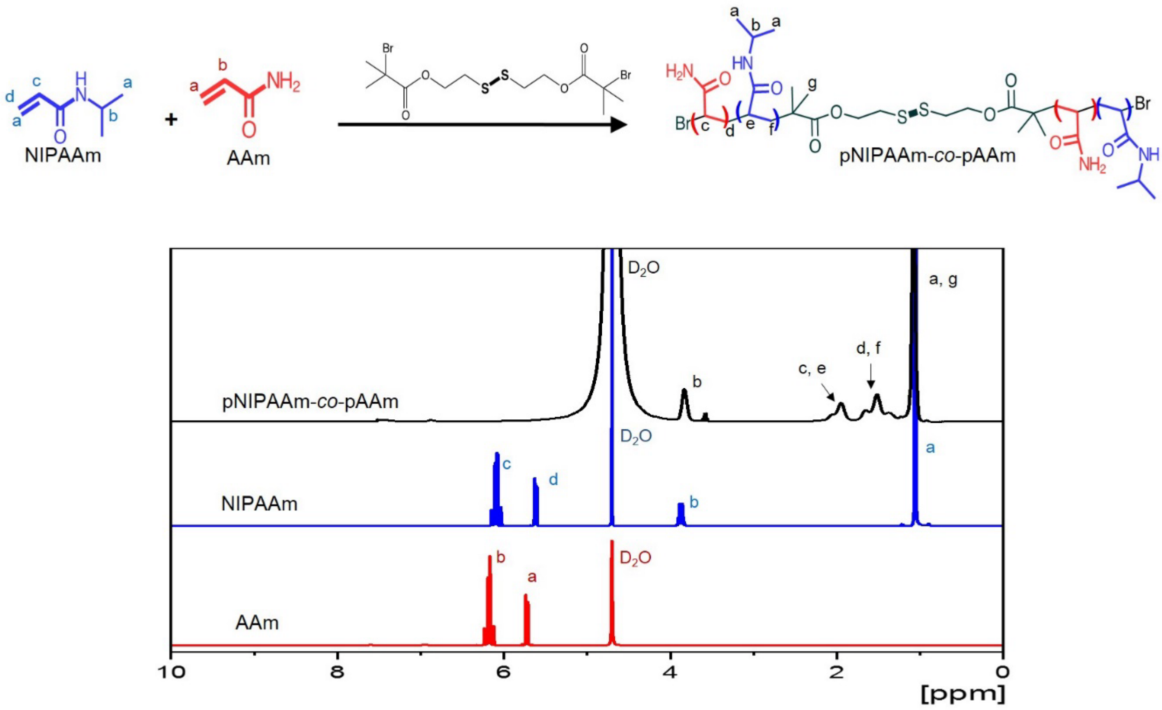

Figure S2: ^{1}H NMR spectra of two monomers, NIPAAm (blue) and AAm (red), and polymer **1** product (black), in D_2O.

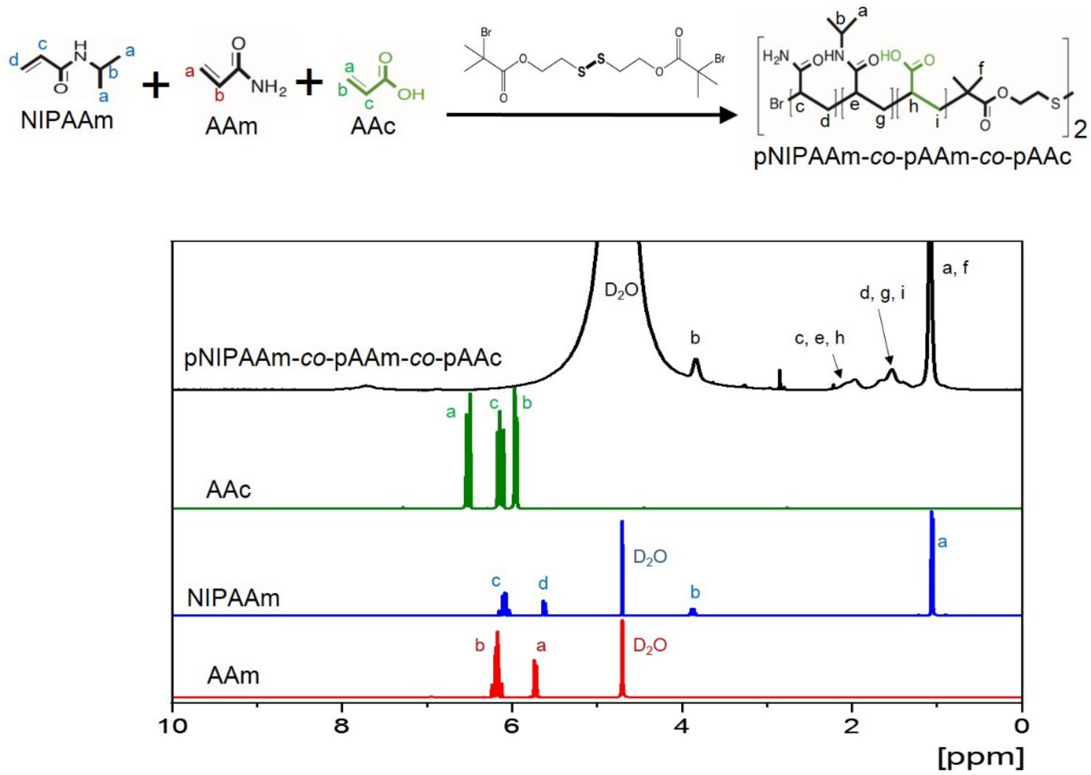

Figure S3: ^{1}H NMR spectra of three monomers, NIPAAm (blue), AAm (red), and AAc (green) and polymer **2** product (black), in D_2O.

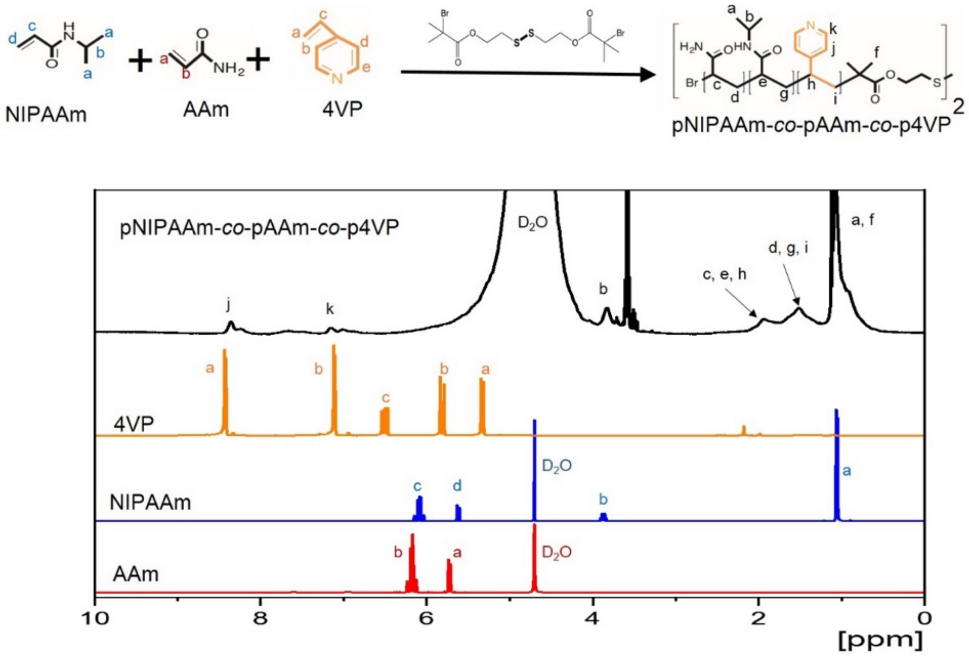

Figure S4: ^{1}H NMR spectra of three monomers, NIPAAm (blue), AAm (red), and 4VP (orange) and polymer **3** product (black), in D_2O.

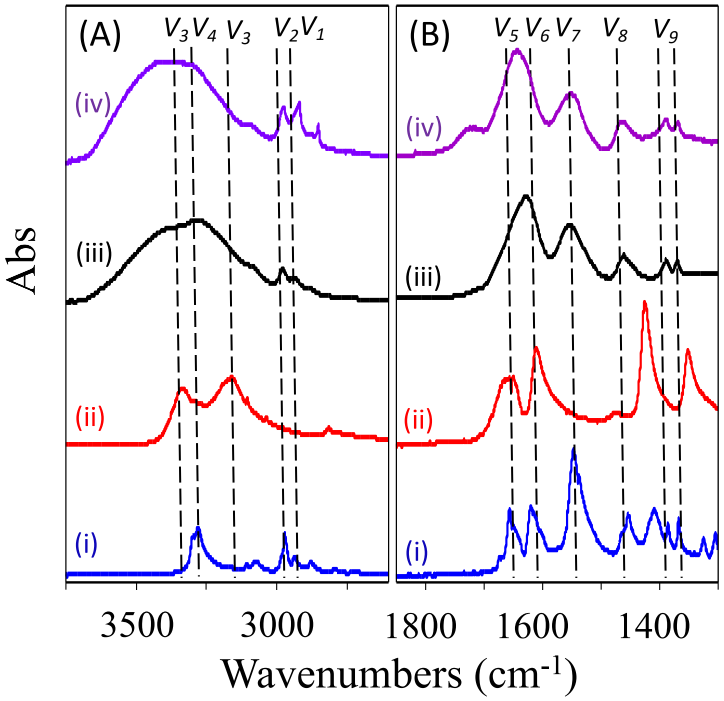

Figure S5: FTIR results characterizing the monomers NIPAAm (i) and AAm (ii), polymer **1** (iii), and the **1**-NP (iv).

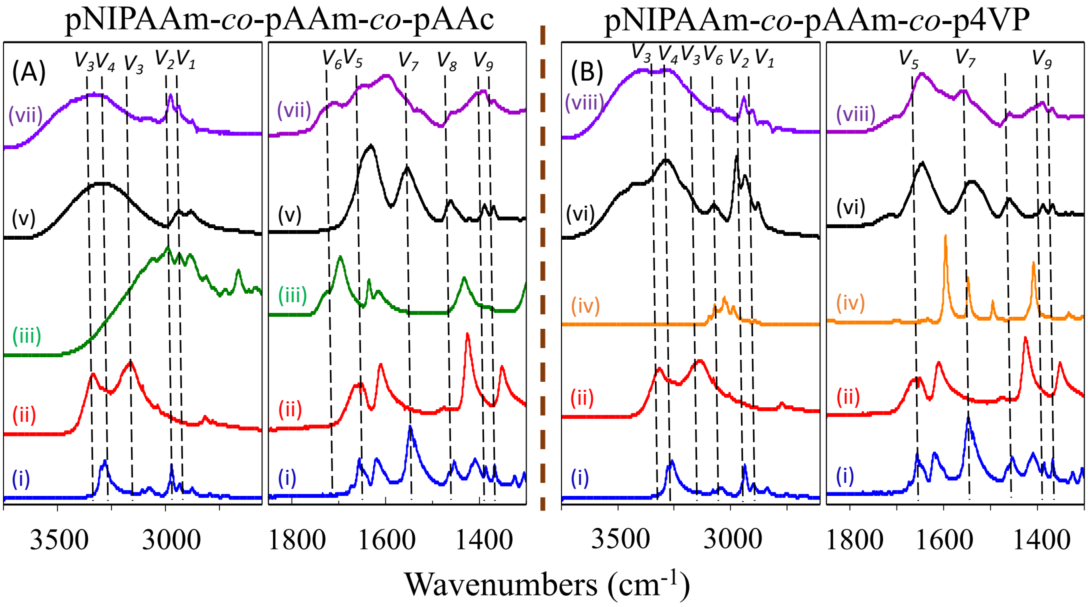

Figure S6: FTIR of NIPAAm (a,b-i) and AAm (a,b-ii), AAc (a-iii), 4VP (b-iv), polymer **2** (a-v), polymer **3** (b-vi) and **2**-NP (a-vii), and **3**-NP (b-viii).

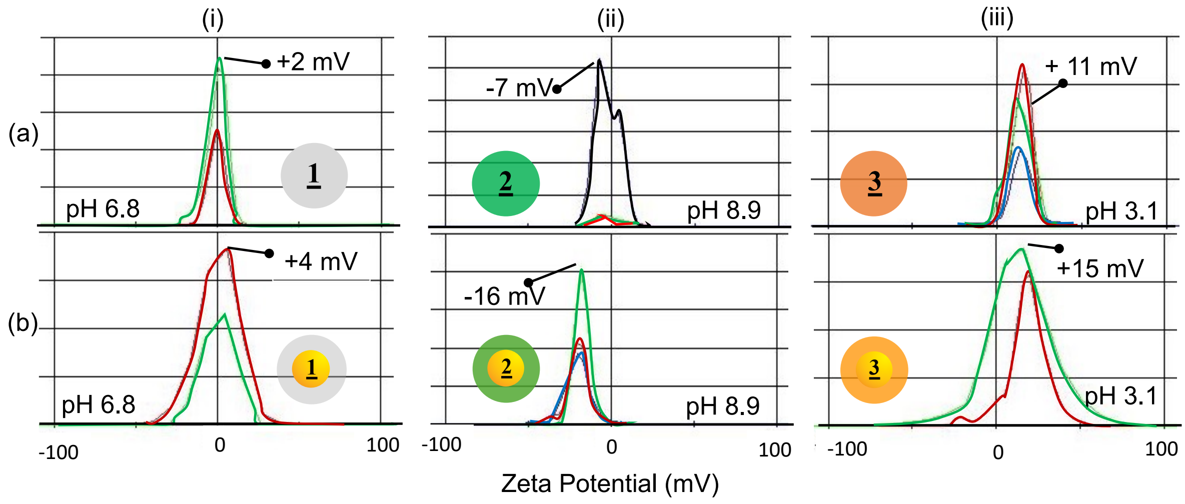

Figure S7: Zeta potential comparisons between polymer- (a), and polymer-NP (b) for **1**- (i), **2**-(ii), and **3**-systems at the pH shown. Colors indicate multiple trials of the same sample, pH adjusted with dilute HCl or NaOH in 10 mM NaCl, T = 25 °C.

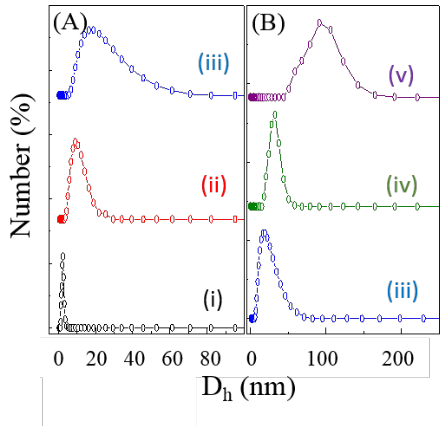

Figure S8: Representative DLS of polymer **1** (a-i) and Au NPs before functionalization (a-ii), and after modification (**1**-AuNP) at T< T_C (a, b-iii), **1**-NP at T > T_C (b-iv, after 15 min) and at T > T_C (b-v, after 1 hr).

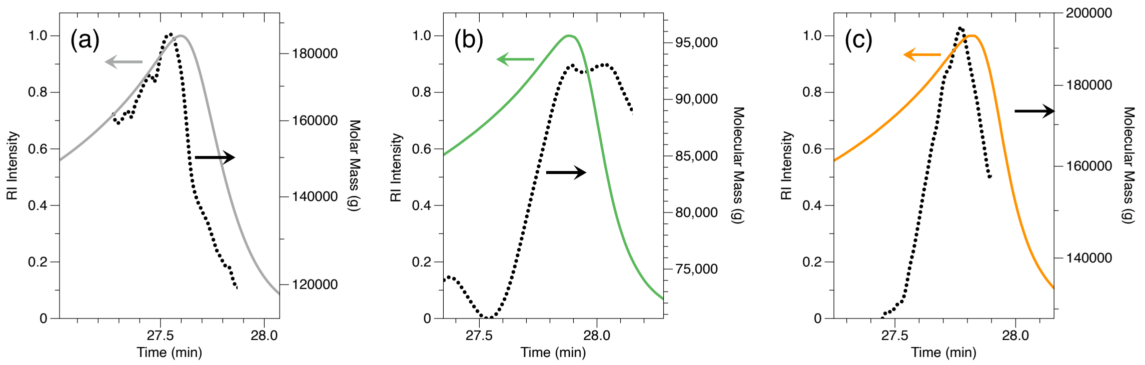

Figure S9: SEC-MALS results for replicate **1**- (a), **2**- (b), and **3**-polymers (c) prepared at [NIPAAm]:[AAm]:[AAc/4VP] = 90: 05: 05 molar ratios. Results show the primary SEC fraction separation coupled with corresponding MALS molecular mass readings. Measured in 1x PBS at 25ºC.

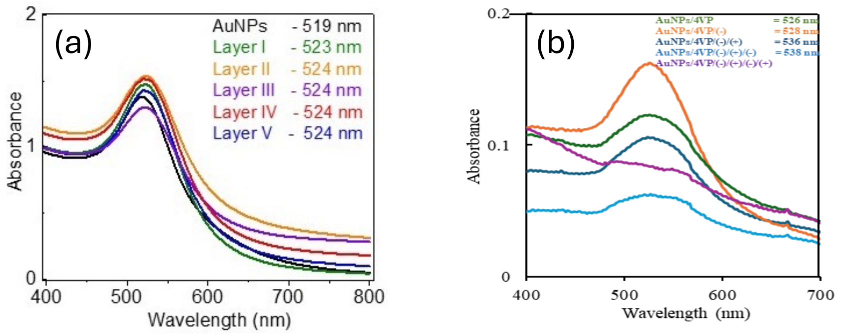

Figure S10: Representative UV-Vis of PE-**2**-AuNP (a) and PE-**3**-AuNP (a), after each PE layer deposition shown. In pure water.

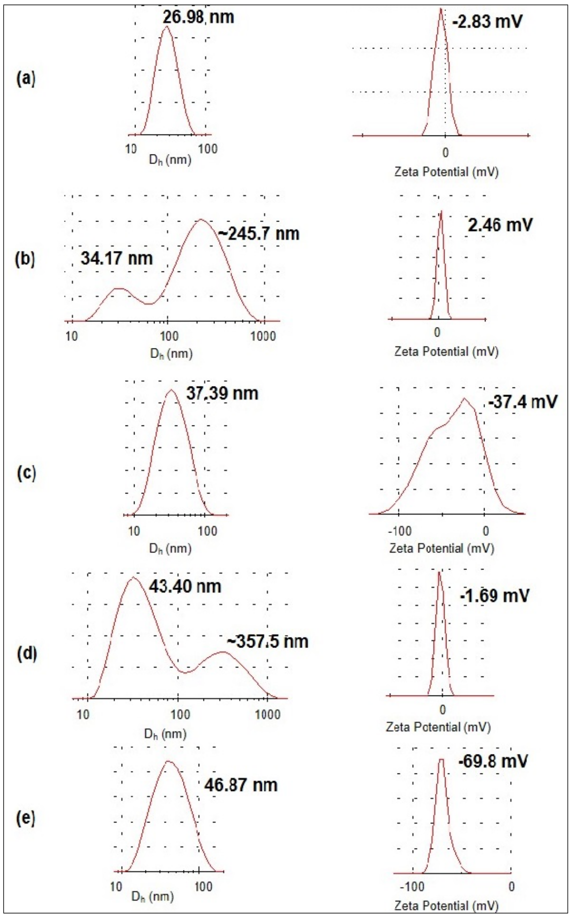

Figure S11: Characterization of PE-2-AuNP, via DLS (left panel), and Zeta Potential (right panel) at deposition layer I (a), layer II (b), layer III (c), layer IV (d), and layer V (e).

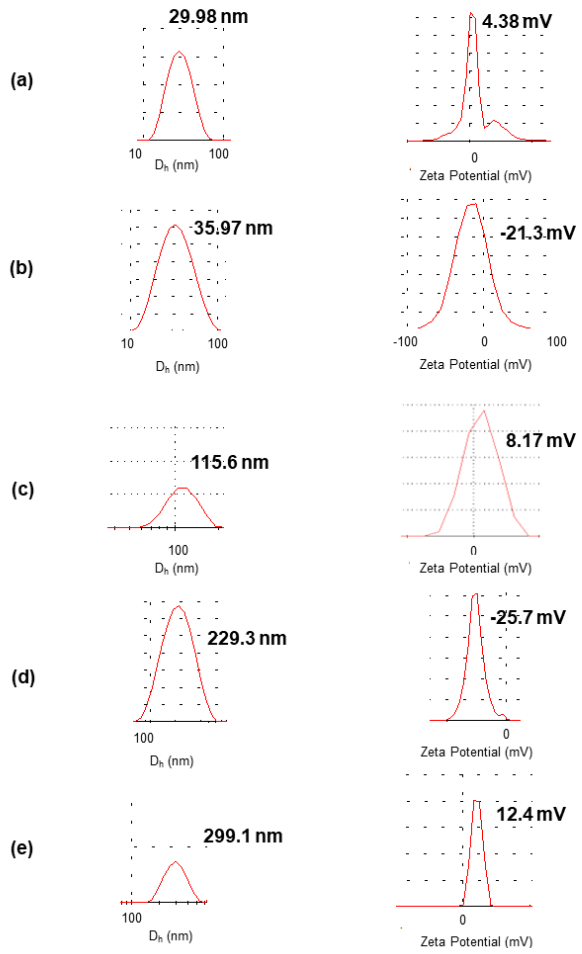

Figure S12: Characterization of PE-3-AuNP, via DLS (left panel), and Zeta Potential (right panel) at deposition layer I (a), layer II (b), layer III (c), layer IV (d), and layer V (e).
